# Socio-psychological characteristics of persons who had attempted suicide (Minsk, Belarus)

**DOI:** 10.1192/j.eurpsy.2022.651

**Published:** 2022-09-01

**Authors:** P. Lapanau, S. Davidovsky

**Affiliations:** 1 Belarusian State Pedagogical University, Institute Of Psychology, Minsk, Belarus; 2 Belarusian Medical Academy for Postgraduate Training, Psychotherapy, Minsk, Belarus

**Keywords:** suicidal and self-harming behavior, suicidal intentions, borderline personality disorder

## Abstract

**Introduction:**

Mortality from intentional self-harm is an urgent medical and social problem in most countries of the world. According to the World Health Organization, suicide mortality is the second leading cause of death among young people aged 15-29 years globally. In the Republic of Belarus, this is one of the main causes of death from external causes.

**Objectives:**

To identify statistically significant social factors and individual characteristics of people who had attempted suicide.

**Methods:**

Three patient groups were formed: persons who had suicidal attempt with high probability of death (mainly hanging) (GSAD), persons who had suicidal attempt in other ways (GSAO), and persons who had diagnosed adjustment disorder and did not have suicidal attempts (comparison group CG). The groups consisted of 40, 80 and 40 people, respectively. Socio-demographic data were determined, the level of stress was assessed according to the Holmes and Rahe Stress Scale, depressive symptoms on the Montgomery-Åsberg Depression Rating Scale, individual features were determined using the Eysenck personality questionnaire and the Leonhard-Shmishek personality characteristics questionnaire. The obtained data were analyzed using the Spearman linear correlation coefficient.

**Results:**

Statistical analysis revealed one significant factor interconnected with a high level of motivation for committing suicide - the method of suicide (rₛ=-0.68) and 2 factors were at the significance boundary: the presence of a diagnosis of mental disorder (rₛ=0.28), and the education factor (rₛ=-0.28).

**Conclusions:**

The method of suicide, the presence of a mental disorder and the level of education are the most significant factors interrelated with a pronounced motivation for committing suicide.

**Disclosure:**

No significant relationships.

